# Length Normalized Indices for Fat Mass and Fat-Free Mass in Preterm and Term Infants during the First Six Months of Life

**DOI:** 10.3390/nu8070417

**Published:** 2016-07-08

**Authors:** Ipsita Goswami, Niels Rochow, Gerhard Fusch, Kai Liu, Michael L. Marrin, Matthias Heckmann, Mathias Nelle, Christoph Fusch

**Affiliations:** 1 Division of Neonatology, Department of Pediatrics, University of Calgary, 780C, Foothills Medical Centre, 1403, 29th Street NW, Calgary, AB T2N 2T9, Canada; ipsita.goswami@ahs.ca; 2 Division of Neonatology, Department of Pediatrics, McMaster University, 1280 Main Street West, Room HSC-4F5, Hamilton, ON L8S 4K1, Canada; nielsrochow@gmail.com (N.R.); gefusch@mcmaster.ca (G.F.); marrin@mcmaster.ca (M.L.M.); 3 Department of Mathematics and Statistics, McMaster University, 1280 Main Street West, Hamilton, ON L8S 4K1, Canada; liuk25@math.mcmaster.ca; 4 Department of Pediatrics, University of Greifswald, Ferdinand-Sauerbruch-Straße, Greifswald 17475, Germany; matthias.heckmann@uni-greifswald.de; 5 Department of Pediatrics, Division of Neonatology, Inselspital and University of Berne, Effingerstraße 102, Berne 3010, Switzerland; mathias.nelle@insel.ch; 6 Department of Pediatrics, Paracelsus Medical University Nuremberg, Nuremberg General Hospital, Prof.-Ernst-Nathan-Str. 1, Nuremberg 90219, Germany

**Keywords:** fat mass index, fat free mass index, body composition, fat mass, lean mass, growth, nutrition, postnatal

## Abstract

Objective: Postnatal tissue accretion in preterm infants differs from those in utero, affecting body composition (BC) and lifelong morbidity. Length normalized BC data allows infants with different body lengths to be compared and followed longitudinally. This study aims to analyze BC of preterm and term infants during the first six months of life. Methods: The BC data, measured using dual energy X-ray absorptiometry, of 389 preterm and 132 term infants from four longitudinal studies were combined. Fat-mass/length^2^ (FMI) and fat-free mass/length^2^ (FFMI) for postmenstrual age were calculated after reaching full enteral feeding, at term and two further time points up to six months corrected age. Results: Median FMI (preterm) increased from 0.4 kg/m^2^ at 30 weeks to 2.5, 4.3, and 4.8 kg/m^2^ compared to 1.7, 4.7, and 6 kg/m^2^ in term infants at 40, 52, and 64 weeks, respectively. Median FFMI (preterm) increased from 8.5 kg/m^2^ (30 weeks) to 11.4 kg/m^2^ (45 weeks) and remained constant thereafter, whereas term FFMI remained constant at 11 kg/m^2^ throughout the tested time points. Conclusion: The study provides a large dataset of length normalized BC indices. Followed longitudinally, term and preterm infants differ considerably during early infancy in the pattern of change in FMI and FFMI for age.

## 1. Introduction

Body composition measurements, particularly longitudinal measurements of lean and fat mass can be used to study the effects of nutrition on growth and developmental programming during the “critical period” of early infant development [1,2,3]. Optimal nutritional intake and adequate weight gain before term age are associated with improved neurocognitive outcomes in preterm infants [4]. However, rapid postnatal weight gain, especially due to fat, increases the risk of central adiposity and cardiovascular diseases [5]. With the improvements in preterm infant survival, the optimization of body composition, cognitive outcomes, and metabolic health is a priority.

Total weight gain, the most commonly used clinical tool for guiding nutritional management in preterms, fails to differentiate between proportionate gain in fat mass and lean mass. Fat-free mass (FFM) and fat mass (FM) considered separately are true estimates of nutritional status of the individual infant when compared to a reference. The use of body weight (BW) for normalization (e.g., %FM as FM/BW or %FFM as FFM/BW) fails to account for independent tissue accretion rates because %FM depends on the amount of FFM and vice versa. This approach is, therefore, not useful in the longitudinal assessment of body composition in neonates who undergo skeletal and muscle growth along with fat accretion. The %FM value underestimates true fat accretion in the presence of increasing FFM and overestimates the fat gain if there is poor tissue growth [6]. To overcome this limitation, height-normalized indices have been used to define nutritional depletion or obesity in adults [6,7,8]. In infants similarly length-normalized FM and FFM indices can potentially be useful in comparing the body compositions of individuals with different lengths, particularly in preterm infants [7,9]. The lower absolute FFM in these infants is partly caused by their shorter body length compared to term infants. The use of the FFMI will eliminate this difference, such that a low FFMI in the preterm will reflect a truly lower FFM. When expressed as percentiles, these indices can determine whether or not an individual falls within the normal range of the population. Van-Itallie proposed using body length to normalize FM and FFM, similar to its use in BMI [6]. The BMI has limitations in the assessment of body fat in neonates and older children [10,11]. Weight gain can be predominantly fat or predominantly lean mass gain in a growing neonate. Length increase is a proxy for skeletal and muscle mass increase, which in turn reflects FFM. Compared with BMI or body fat percentage, FMI has higher sensitivity for slight changes in body fat stores, making it a superior method for assessing static and dynamic nutritional status. FMI can identify subjects with “normal” BMI but who are at potential risk because of elevated FM. The body proportionality index is gaining attention in the assessment of growth and nutrition of premature infants [12,13]. However, it is not yet in wide use because reference standards have yet to be defined [8].

As body composition measurements are yet to become a part of bedside practice, reference data for newborns and infants are scarce. It was therefore the aim of this study to develop growth charts for length normalized body composition indices for preterm and term infants from birth until six months corrected age.

## 2. Material and Methods

### 2.1. Subjects

Data were combined from 4 longitudinal studies involving a total of 521 infants, 22 to 42 weeks of gestation, from a predominantly Northern European descent population. Infants with major congenital, chromosomal, or metabolic anomalies and multiple births other than twins were excluded. Infants with severe chronic lung disease, sepsis, and NEC were also excluded. Infants were fed using the same feeding protocol in both units. During the study measurements, they were on full enteral feeds (150 to 170 mL/kg/day) with fortified breast milk or formula (80 kcal/dL) for preterm and breast milk or formula (67 kcal/dL) for term infants. Body composition measurements were taken after the infants had reached full enteral feeds and were clinically stable and growing at term corrected gestation and two further occasions up to a corrected age of 6 months. Post-feed measurements were obtained in a quiet, warm room during sleep, with the infants swaddled in cotton blankets without additional clothing. The details of the included studies are summarized in Table 1.

### 2.2. Ethics Approval

All studies were approved by local University Ethical Committee and the German studies were approved by the State Authority for Radiation Exposure and Control. Informed consent was obtained from parents prior to performing the measurement. Ethics Approval codes for Study 1 SNF# 3200-43586, study 2 is REB#: UV 07/01, study 3 is REB#: UV 26/98, study 4 is REB#: UV 70/03. Study 4 was registered with clinicaltrials.gov as NCT00196482.

### 2.3. Anthropometry

Weight was measured to the nearest 10 g by using a standard beam balance. Body length and head circumference (HC) were measured in triplicate to the nearest 0.5 and 0.1 cm, respectively. Body length was measured using a measuring board (Schäfer, Karlsruhe, Germany). HC was measured using a standard tape.

### 2.4. Dual Energy Xray Absorptiometry (DEXA)

Body composition was measured using a whole body scanner (QDR 1500, Hologic, Waltham, MA, USA) in single-beam mode. The X-ray tube was pulsed between high and low voltage (140 or 70 kV) at a rate of 50 Hz to produce dual-energy X-ray beams. A detector mounted above the infant measured the transmitted intensity on a pixel-by-pixel basis. External calibration was performed with a step phantom with known equivalent amounts of fat and lean tissue. Daily quality control scans were done using a manufacturer-supplied anthropometric spine phantom. For ethical reasons, duplicate measurements were not performed. All scans were performed on an infant platform with the infant in supine position by a whole-body scan procedure. The platform filtered the low-energy beam to improve system linearity in the small subjects and reduced the radiation dose. The scan time was approximately 5 min. The scans were analyzed using modified infant whole-body software (version 5.67, Hologic, Waltham, MA, USA). The device had been previously calibrated for neonatal body composition using chemical analysis in a sophisticated piglet model [14]. Results included body skeletal area (cm^2^), bone mineral content (g), fat mass (g), lean mass (g), and percentage body fat (%). Fat free mass (FFM) is the sum of bone mineral content and lean mass.

### 2.5. Data Analysis

To control for variation at the right tail due to missing values by dropout imputation was applied to replace missing data with plausible values predicted from regression models based on the observed data. The regression model used for our data is a linear mixed-effect model, given by (1)

(1) $$Y_{it}=\;\underset{fixed\;effect}{\underset{︸}{\alpha_{0}+\alpha_{1}age+\alpha_{2}age^{2}}}+\underset{random\;effect}{\underset{︸}{\beta_{i0}+\beta_{i1}age}}+\epsilon_{it},$$

where *i* is the infant, *t* is the measurement, and *Y* is any longitudinal variable with missing data. The fixed effect represents the regression model for the population, while the random effect indicates that for each infant. The individual regression curves are allowed to deviate from the population regression curve with an intercept $\beta_{i0}$ and a slope $\beta_{i1}$. Coefficients are estimated with observed data and lead to a single set of estimates ${\hat{\alpha }}_{0},\;{\hat{\alpha }}_{1},\;{\hat{\alpha }}_{2}$ for $\alpha_{0},\alpha_{1},\alpha_{2}$ and *n* sets of estimate ${\hat{\beta }}_{i0},\;{\hat{\beta }}_{it}$ for $\beta_{i0},\beta_{i1}$, where *n* is the number of infants. With the estimate of coefficients, we can obtain the predictive model (2):

(2) $${\hat{Y}}_{it}=\;{\hat{\alpha }}_{0}+{\hat{\alpha }}_{1}age+{\hat{\alpha }}_{2}age^{2}+{\hat{\beta }}_{i0}+{\hat{\beta }}_{it}age,$$

from which the plausible values of the missing data can be calculated. By using this model, each infant has its own regression model (curve) so that the variances are reduced compared with a simple linear model.

### 2.6. Growth Modeling

We used Generalized Additive Models for Location, Scale and Shape (GAMLSS) to estimate age-dependent percentile curves for all parameters. GAMLSS was one of the methods evaluated for developing the WHO child growth standards [15]. GAMLSS is a semi-parametric univariate regression model that allows flexible modeling of both the distribution of the dependent variable and the dependence of all parameters of the distribution on explanatory variables [16]. The parameters are denoted by μ (location), σ (variance), ν (skewness), τ (kurtosis), and λ (power transformation of explanatory variable). The distribution of the response variable was chosen from the Box-Cox *t* distribution family and cubic splines as a smoothing technique. An automated iterative optimization process was used to find the degrees of freedom and fit the model by maximizing penalized likelihood [17].

## 3. Results

Among 521 infants recruited, 132 were terms, and 389 were preterms. Each observation was regarded as an independent data point for construction of the growth curves. We had a total of 858 measurements including 571 data points from preterm and 287 from term infants. The percentage of infants who had two, three, and four repeated observations were 17%, 21%, and 3% respectively. To compensate for the lower percentage of infants who had multiple measurements, we imputed our data as described in the Materials and Methods section. For term infants, we used imputation so that they had at least three measurements and, for preterm infants, at least four measurements. A mixed effect model was used to create the individual models so that the prediction was based on a subject-specific level. Thus, the infants who had only one measurement could not be predicted because of the limited information. After imputation, 1383 observations in preterm and 399 in term infants were used for the final data analysis. Median body weight and length of preterm infants remained consistently lower than term infants at the same postmenstrual age (PMA) (Table 2). The head circumferences of the preterm infants approached those of the term infants by 60 weeks PMA.

Length in preterm infants increased by 0.84 cm/week and in term infants by 0.76 cm/week, but preterm infants remained shorter than did terms. Preterm HC increased by 0.52 cm/week, slightly higher than 0.41 cm/week in terms. Median length of preterm infants at 28, 38, 50, 60, and 70 weeks PMA was 36 cm, 46 cm, 56 cm, 63 cm, and 69 cm, respectively. Term infants had a median length of 49cm, 59 cm, and 66 cm at 38, 50, and 60 weeks PMA. The median centile for the head circumference in term infants increased from 34 cm at 38 weeks to 39 cm (50 weeks) and 42 cm (60 weeks). The corresponding centile in preterm infants was 25 cm, 33 cm, 39 cm, 43 cm, and 46 cm at 28, 38, 50, 60, and 70 weeks PMA, respectively.

**Figure 1:**
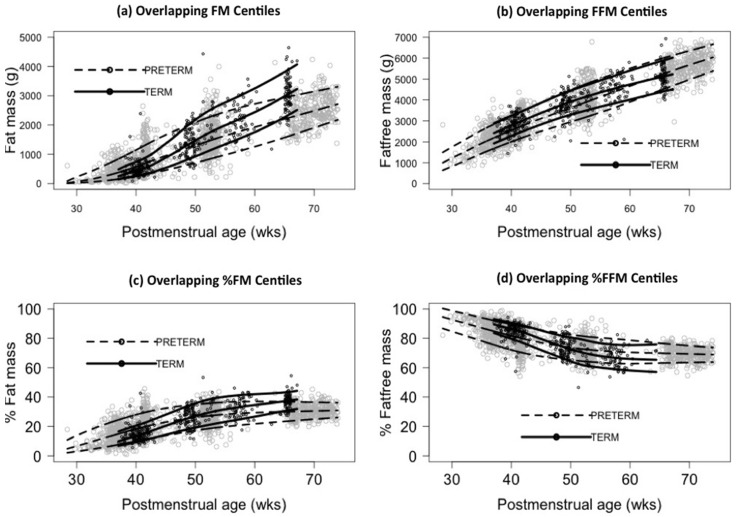
Overlapping 10th, 50th, and 90th centiles of FM and FFM in term and preterm infants. Preterm infants gained FFM faster than FM until corrected term. Beyond 50 weeks PMA, the rate of increase in FM was slower in preterms compared with terms. Preterm infants caught up with term FFM by 65 weeks PMA. (**c**,**d**) Overlapping 10th, 50th, and 90th centiles of %FM and %FFM over age in preterm and term infants. As age progressed, %FM increased, and %FFM decreased at the same rate. Until term equivalent, preterm infants had faster increases in %FM than term infants but subsequently slowed down and beyond 50 weeks, term infants had a faster increase until 6 months corrected age.

From birth to 50 weeks PMA, preterm infants acquired FM faster than term infants, beyond which the gain in FM was less than that for term infants (Figure 1a). Until term equivalent age (TE), preterm infants gained FFM over time faster than the gain in FM. Preterm infants started with a lower FFM at birth but caught up with term FFM centiles by 65 weeks PMA (Figure 1b). Both term and preterm infants slowed down their tissue accretion rate beyond three months PMA. With increasing PMA all infants in both groups had an increase in %FM and a corresponding decrease in %FFM. The rate of change was faster in preterms until TE but subsequently slowed down compared with terms (Figure 1c,d).

**Figure 2:**
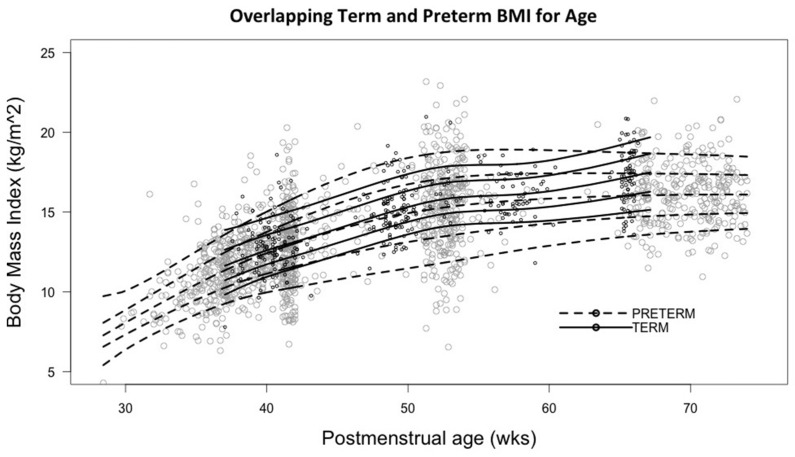
10th, 25th, 50th, 75th, and 90th centiles of BMI for postmenstrual age in term and preterm infants based on the imputed data set.

The median BMI of preterm infants increased from birth (8 kg/m^2^) to 50 weeks PMA and then remained constant around 15 kg/m^2^ up to six months corrected age (Figure 2). Term infants were born with a median BMI of 13 kg/m^2^ and achieved up to 16 kg/m^2^ by early infancy. The BMI in preterm infants increased linearly with %FM (*r*^2^ = 0.55), FM (*r*^2^ = 0.58), FFM (*r*^2^ = 0.65), and bone mineral content (*r*^2^ = 0.57). In term infants, BMI increased linearly with %FM (*r*^2^ = 0.65), FM (*r*^2^ = 0.72), FFM (*r*^2^ = 0.63), and bone mineral content (*r*^2^ = 0.72).

**Figure 3:**
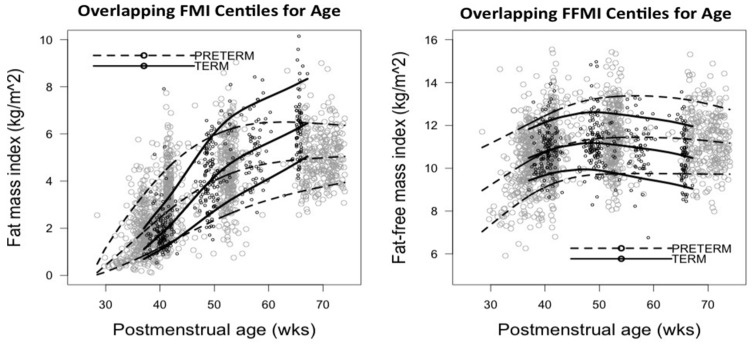
10th, 50th, and 90th centiles of FMI and FFMI for postmenstrual age in term and preterm infants based on the imputed data set.

**Figure 4:**
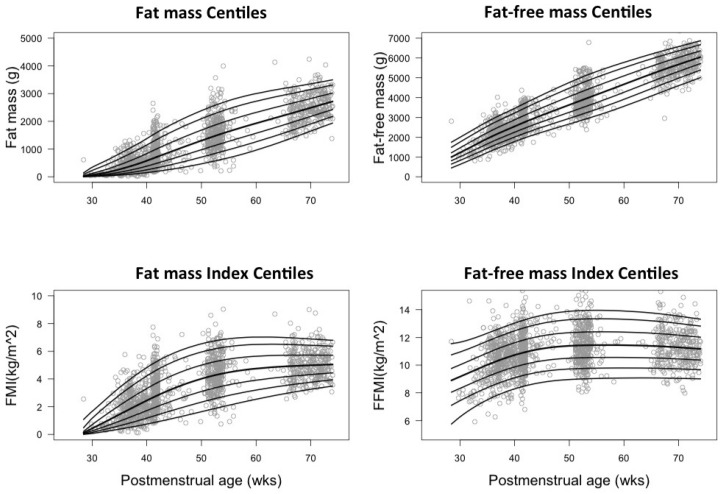
Growth curves showing 3rd, 10th, 25th, 50th, 75th, 90th, and 95th centiles for preterm infants constructed from imputed dataset.

**Figure 5:**
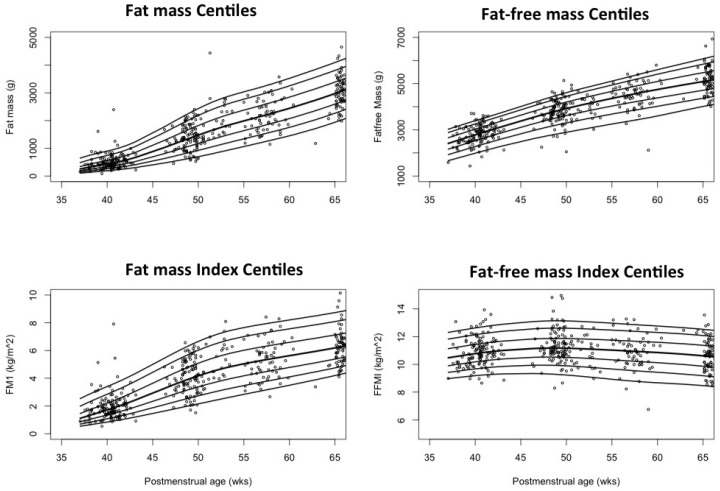
Growth curves showing 3rd, 10th, 25th, 50th, 75th, 90th, and 95th centiles for term infants constructed from imputed dataset.

The median FMI for preterm infants increased from 0.4 kg/m^2^ at 30 weeks to 2.5, 4.3, and 4.8 kg/m^2^ at 40, 52, and 64 weeks, while term infants measured 1.7, 4.7, and 6 kg/m^2^ at 40, 52, and 64 weeks, respectively (Tables S1 and S2). The median FFMI in preterm infants increased between 30 and 45 weeks from 8.5 to 11.4 kg/m^2^ but remained constant thereafter until 64 weeks. The term median FFMI remained constant throughout the tested time points (10–11 kg/m^2^). A rapid increase in FMI and FFMI was noted in preterm infants from birth until TE, the latter being faster. FMI of both term and preterm infants increased with age until around 50 weeks, the rate of increase in preterms being much higher than terms. Subsequently, both groups slowed down indicated by the flattening of the curves (Figure 3). Percentile curves for FM, FMI, FFM, and FFMI were plotted against PMA from the imputed data set (Figure 4 and Figure 5).

## 4. Discussion

In the current study, we present longitudinal length normalized body composition data from a large dataset of stable growing term and preterm infants on standard feeding regimens during the first six months of life. Normal tissue accretion in utero is interrupted by preterm birth, and this data provides important comparative information about body composition of term and preterm infants with the same PMA. Currently, the optimum body composition of preterm infants is not known but adaptive processes transitioning IGF-2 controlled growth to IGF-1 might cause postnatal growth trajectory to deviate from intrauterine growth. The first six months of postnatal life are a critical period for developmental programming, and our study reflects changes in body composition during this period. BMI curves have been recently introduced for preterm growth assessment, but our data suggest that length normalized FMI and FFMI used in conjunction with a growth curve derived from a representative population can be a more meaningful tool to assess nutritional status in neonates especially in preterm infants.

Several studies report normative data on body composition in term infants from birth to six months of age [18,19]. However, there is still a paucity of longitudinal reference data on body composition in preterm infants. We present data of FMI and FFMI from a dataset comprising >1000 data points. In previous studies, weight-normalized parameters such as %FM have been used to compare body composition of preterm infants at corrected term, three and five months corrected age, with exclusively breastfed term infants [20]. We found that %FM and %FFM represent reciprocal changes in body compartments and hence are not independent parameters. FMI and FFMI represent the change in the independent body compartments relative to length and can be used for longitudinal growth assessment. Enhancing nutrition to replicate in-utero changes in body composition has been a goal despite the fact that nutrient accretion is different ex-utero [21,22]. The ideal preterm growth pattern is still unknown. Contrary to intrauterine growth curves, which show cross-sectional fetal growth rather than longitudinal growth of ex-utero preterm infants [23], our data illustrate postnatal growth over time and how it varies from term infants. Reference values for the composition of postnatal growth can be used to study the effects of nutritional and long-term outcomes. Extra-uterine growth restriction is a modifiable risk factor of adverse neonatal outcome that can be missed if a proper assessment tool is not used [12]. A significant association exists between poor linear growth and neurodevelopmental outcome at 2 years in infants with a low birth weight [24]. In a cohort < 2500 g, early (term to 4 months) linear growth was associated with better IQ at 8 and 18 years. In contrast, BMI gain during the same period did not appear to benefit cognition, but rather increased the odds of obesity [5]. Our study describes the qualitative nature of growth during this critical period.

Weight for age is considered to be a poor tool to assess growth when weight gain is disproportionate with linear growth [12]. We have previously reported that categorization according to birth weight centiles fails to reflect body fatness in newborns [25]. Body proportionality indices such as Lubchenco’s weight for length charts and the Rohrers Ponderal index have been used to assess nutrition, but there are several limitations to these [12,23,26]. Although used routinely in children as a measure of adiposity, BMI has not been widely applied to preterm infants. Two recent studies added reference BMI for age growth curves to quantify growth in preterms between 22 to 42 weeks GA [13,27]. Earlier studies have noted a weak association between the BMI *z*-score of term infants and %FM (*r*^2^ = 0.43) [10] and the BMI of preterm infants at TE with FM (*r*^2^ = 0.47) and %FM (*r*^2^ = 0.34) [28]. We found that the BMI of term infants had a slightly better correlation (*r*^2^ = 0.7) with %FM and FM than the BMI of preterm infants (*r*^2^ = 0.5). Compared with terms, the correlation between BMI and bone mineral content is also poor. However, there is a good correlation (*r*^2^ = 0.72) between BMI and FFM irrespective of gestational age at birth. This is likely, because neonates gain both FM and FFM simultaneously as they grow, and there is a substantial difference in the proportion of these compartments at different PMAs. BMI alone is insufficient in assessing the gain of FM and bone growth in preterm infants, whereas FMI and FFMI may be informative.

From birth to TE, preterm infants gain FFM faster than FM. When considered in relation to overall weight gain, %FM increases, while %FFM decreases reciprocally. The faster gain in FFM relative to linear growth is evident from a steeper curve of FFMI compared to FMI over time. Two studies have published BMI curves in preterm infants, but there is no previous data on the change in FMI/FFMI with age in preterms. The initial rapid increase of FFMI supports the importance of aggressive nutrition to prevent protein and energy deficit. The more immature the infant is at birth, the higher is the protein: energy (PE) ratio needed to meet the goal of adequate weight gain relative to fat [29]. Providing the optimal nutrition in the form of a higher PE ratio before TE is important in programming these infants for optimal growth patterns.

At TE, preterm infants in our study were noted to have higher absolute FM than terms, but a lower length, HC, and FFM. A meta-analysis of eight studies that used recognized techniques of body composition measurements concluded that preterm infants at TE had greater %FM but significantly less FM than term infants at birth, in the methodology subgroup analysis of studies done with DEXA there was no difference in FM between the two groups [30]. Roggero et al. used air displacement plethysmography and demonstrated, similar to our results, that mean FM value at term for all preterm infants, regardless of intrauterine and postnatal growth restriction, was much higher than that found in full-term neonates at birth [20]. Contrary to our results, however, they found that the mean FM values attained at three and five months by all infants were comparable to those of full-term breastfed infants. In our preterm cohort, beyond TE, the gain in %FM and FMI is slower in preterms compared with terms; thus, they have less absolute FM than do terms. It has been previously noted that preterm infants continue to have less FM than term infants until eight years, although the distribution has a propensity towards central adiposity [9]. Unfortunately, the method of body composition measurement we used, DEXA, is unable to demarcate the distribution of the fat in our cohort. There have been concerns that preterm infants at TE have higher %FM than term infants at birth. Our data confirms this finding.

The preterm cohort in our study did not achieve the same in utero length and FFM at TE as term infants at birth, although they were a relatively healthy population on a standard diet, and the unit policy for nutrition was to avoid postnatal growth retardation. We used an up-to-date feeding regimen and assessment of growth and evaluation of nutrition was an integral part of daily rounds. In this respect, our data constitute normative distribution data. The observed differences between the growth of preterm and term infants may reflect a reduced longitudinal growth potential as a reaction to the switch from intra- to extra-uterine environment, as the optimal dietary regime for longitudinal growth has not yet been determined. This hypothesis is supported by the recent observation that unstandardized breast milk composition [31] and inappropriately balanced carbohydrate-fat ratios impact longitudinal growth despite an appropriate energy content [32]. FFMI hardly varies in neonates beyond TE, but preterm infants have higher FFMI than terms with the same PMA. Thus, preterm infants gain more muscle and lean mass relative to length compared with terms.

In comparing the body composition centiles of our term cohort to reference data published from an Ethiopian cohort of healthy term infants, we noted a similar pattern of change in the first half of infancy, but our FMI centiles were higher [33]. This difference may be attributed to difference in nutritional standards of the population and the method used for body composition measurement (air displacement plethysmography vs. DEXA). The longitudinal change in median FM and FFM in our term cohort with age are consistent with mean values of previously published FM and FFM in exclusively breastfed term infants [18].

Our study had several limitations. Measuring body composition in the clinical setting is expensive, skill-based, and requires sophisticated equipment. Radiation exposure with DEXA makes it unsuitable for repeated measurements. The data points were not equally distributed over the observation period. The density of data points used in our study to plot the growth curves is higher in the first 3 months. Beyond 60 weeks, especially in term infants, less data points are available, so the interpretation is guarded. We tried to compensate for the low number of infants with multiple measurements by using imputation in our analysis. The current study was not designed to investigate the association between body composition in the first six months and later morbidity or neurodevelopmental outcome. Measurement of length in infants is challenging due to the need for extension of limbs. We did not include variables such as maternal BMI and postnatal factors that might affect growth in early infancy. Our data analysis does not differentiate grown infants from growth-restricted infants appropriately. Thus, it would be difficult to derive whether the growth trajectory in each sub-population is different. The longitudinal change in body composition, especially %FM, can be different in AGA and SGA preterm infants [20]. It may therefore be beneficial in future studies to address the difference in FMI and FFMI in two groups of preterm infants. We have not presented gender-specific FMI and FFMI growth curves, but preterm BMI for age curves were similar in males and females [13,27]. The median BMI changes of our preterm cohort matched the changes noted in Olsen’s preterm BMI curves [13], and the changes in the term cohort were similar to the WHO child growth standard BMI curves for 0–2 years of age. Finally, a limitation of our study is that the analysis was not stratified for feeding either breast milk or formula during the post-discharge period. Body composition is known to be affected by the mode of feeding.

Standardization of growth rates in preterm infants is needed. One multicenter, prospective study on 26- to 37-week-old infants concluded that human growth across different ethnic groups is similar and generalizable despite socioeconomic constraints [34]. The authors also noted that the anthropometry of healthy preterms merged with the measurements of the WHO Child Growth Standards at only 64 weeks PMA. This supports the concern that expecting preterm infants to grow like fetuses, despite postnatal stresses and higher metabolic demand, will result in overfeeding. While prior studies have prescriptive growth standards for anthropometry and BMI in preterm infants, our study provides direct measurements of tissue masses in the same population at the critical time period. Prescriptive body composition percentile charts for preterm infants are needed for present day clinical practice. Similar to the approach that the WHO has used to develop growth charts, we analyzed a selected group of healthy preterm infants fed using standardized feeding guidelines.

## 5. Conclusions

We propose the use of FMI and FFMI centiles to standardize the evaluation of nutritional status in preterm infants. Measuring fat and lean mass in individual infants cannot give a true assessment of the nutritional status unless the linear growth is accounted for. FMI and FFMI are independent indices that allow longitudinal assessment of the change in fat and lean mass separately and thus help in the optimization of nutritional interventions. Knowledge of the expected changes in body composition of healthy preterm infants in early infancy can help us set more realistic goals for growth. Our study provides normative reference data for well preterm infants compared with term infants in the first six months of life. Future studies are needed to establish the value of longitudinal changes in body composition in predicting metabolic risk and neurodevelopmental outcome. It will also be interesting to investigate how nutritional management can be tailored to attain the desired changes in body composition.

## Figures and Tables

**Table 1:** Studies included for analysis of body composition (BC).

Study	Type	Sample Size	Location	Objectives	Data
1	O	111	Perinatal Center of Berne, Switzerland	Aimed to establish normative longitudinal data on body composition in term and preterm infants	Longitudinal growth and BC data were obtained from clinically stable preterm (at full feeds, term, 3 and 6 months PMA) and term infants (at term, 3 and 6 months PMA)
2	O	248	Perinatal Center of Greifswald, Germany	All infants (BW < 1500 g) included as part of a routine protocol for the management of calcium and phosphate supplementation and monitoring of bone mineral content	Three BC measurements were obtained once the infants were at full feeds, at term age and at 3 months PMA
3	I R	113	Perinatal Center of Greifswald, Germany WANT study	Growth and BC during the first four months of life in term and late preterm infants	Late preterm (>34 weeks) and term infants were randomized to receive either to a standard term formula or a term formula supplemented with nucleotides over the first four months of life. Term infants on breast milk served as control. BC was measured during first week of life, at 2 and 4 months of age
4	I	49	Perinatal Center of Greifswald, Germany	Growth and BC in preterm infants who received two different commercially available human milk fortifiers at recommended dosages	Three BC measurements were obtained once the infants were at full feeds, at term age and at 3 months PMA

O—Observational Study, I—Interventional study, R—Randomized.

**Table 2:** Longitudinal change in anthropometric parameters.

PMA	<40		40–50		50–60		>60	
(Weeks)	Term	Preterm	Term	Preterm	Term	Preterm	Term	Preterm
*n* = 858	45	333	135	127	85	85	22	26
Weight (g)	3016	2550	3926	3234	6503	5742	7721	6915
	(2763–3462)	(2136–2801)	(3402–4961)	(2797–4067)	(5993–7197)	(5076–6352)	(7070–8701)	(6371–7830)
Length (cm)	49	45	53	49	63	60	67	66
	(48–50)	(43–47)	(51–57)	(47–52)	(61–65)	(57–61)	(65–70)	(64–69)
Head Circumference (cm)	34	33	37	35	41	40	41	41
	(33–35)	(32–34)	(35–39)	(34–37)	(40–42)	(38–41)	(39–42)	(38–42)
Fat mass (g)	408	324	639	541	2012	1514	2634	1892
	(306–533)	(217–453)	(439–1199)	(355–858)	(1586–2487)	(1196–2003)	(2105–3322)	(1562–2290)
Fat-free mass (g)	2556	2154	3191	2619	4381	4016	4860	4880
	(2436–2844)	(1852–2369)	(2827–3655)	(2382–3057)	(3982–4734)	(3552–4338)	(4458–5240)	(4432–5209)
BMI (kg/m^2^)	12.6	12.1	13.8	14	16.2	16.1	17.2	16.3
	(11.5–13.5)	(10.8–13.2)	(12.7–15.0)	(12.5–15.3)	(15.3–17.2)	(14.8–17.1)	(16.4–18.2)	(14.5–16.7)

Median (IQR) of anthropometric parameters currently used in clinical practice at different postmenstrual ages when body composition measurements were taken, comparing infants who were either terms or preterms at birth.

## Supplementary Materials

The following are available online at http://www.mdpi.com/2072-6643/8/7/417/s1, Table S1: Length normalized indices in preterm infants, Table S2: Length normalized indices in term infants.

## References

[1] Wells J.C., Chomtho S., Fewtrell M.S. (2007). Programming of body composition by early growth and nutrition. Proc. Nutr. Soc..

[2] Rochow N., Fusch G., Muhlinghaus A., Niesytto C., Straube S., Utzig N., Fusch C. (2012). A nutritional program to improve outcome of very low birth weight infants. Clin. Nutr..

[3] Gillman M.W. (2010). Early infancy as a critical period for development of obesity and related conditions. Nestle Nutr. Workshop Ser. Pediatr. Program.

[4] Lucas A. (2005). Long-term programming effects of early nutrition—Implications for the preterm infant. J. Perinatol..

[5] Belfort M.B., Gillman M.W., Buka S.L., Casey P.H., McCormick M.C. (2013). Preterm infant linear growth and adiposity gain: Trade-offs for later weight status and intelligence quotient. J. Pediatr..

[6] VanItallie T.B., Yang M.U., Heymsfield S.B., Funk R.C., Boileau R.A. (1990). Height-normalized indices of the body’s fat-free mass and fat mass: Potentially useful indicators of nutritional status. Am. J. Clin. Nutr..

[7] Kyle U.G., Piccoli A., Pichard C. (2003). Body composition measurements: Interpretation finally made easy for clinical use. Curr. Opin. Clin. Nutr. Metab. Care.

[8] Schutz Y., Kyle U.U., Pichard C. (2002). Fat-free mass index and fat mass index percentiles in Caucasians aged 18–98 years. Int. J. Obes. Relat. Metab. Disord..

[9] Fewtrell M.S., Lucas A., Cole T.J., Wells J.C. (2004). Prematurity and reduced body fatness at 8–12 years of age. Am. J. Clin. Nutr..

[10] De Cunto A., Paviotti G., Ronfani L., Travan L., Bua J., Cont G., Demarini S. (2014). Can body mass index accurately predict adiposity in newborns?. Arch. Dis. Child. Fetal Neonatal Ed..

[11] Fusch G., Raja P., Dung N.Q., Karaolis-Danckert N., Barr R., Fusch C. (2013). Nutritional status in sick children and adolescents is not accurately reflected by BMI-SDS. J. Am. Coll. Nutr..

[12] Olsen I.E., Lawson M.L., Meinzen-Derr J., Sapsford A.L., Schibler K.R., Donovan E.F., Morrow A.L. (2009). Use of a body proportionality index for growth assessment of preterm infants. J. Pediatr..

[13] Olsen I.E., Lawson M.L., Ferguson A.N., Cantrell R., Grabich S.C., Zemel B.S., Clark R.H. (2015). BMI curves for preterm infants. Pediatrics.

[14] Fusch C., Slotboom J., Fuehrer U., Schumacher R., Keisker A., Zimmermann W., Moessinger A., Boesch C., Blum J. (1999). Neonatal body composition: Dual-energy X-ray absorptiometry, magnetic resonance imaging, and three-dimensional chemical shift imaging versus chemical analysis in piglets. Pediatr. Res..

[15] Borghi E., de Onis M., Garza C., Van den Broeck J., Frongillo E.A., Grummer-Strawn L., Van Buuren S., Pan H., Molinari L., Martorell R. (2006). Construction of the World Health Organization child growth standards: Selection of methods for attained growth curves. Stat. Med..

[16] Rigby R.A., Stasinopoulos D.M. (2006). Using the Box-Cox *t* distribution in GAMLSS to model skewness and kurtosis. Stat. Model..

[17] Rigby R.A., Stasinopoulos D.M. (2013). Automatic smoothing parameter selection in GAMLSS with an application to centile estimation. Stat. Methods Med. Res..

[18] Fields D.A., Gilchrist J.M., Catalano P.M., Gianni M.L., Roggero P.M., Mosca F. (2011). Longitudinal body composition data in exclusively breast-fed infants: A multicentre study. Obesity.

[19] Fomon S.J., Haschke F., Ziegler E.E., Nelson S.E. (1982). Body composition of reference children from birth to age 10 years. Am. J. Clin. Nutr..

[20] Roggero P., Gianni M.L., Liotto N., Taroni F., Orsi A., Amato O., Morlacchi L., Piemontese P., Agosti M., Mosca F. (2011). Rapid recovery of fat mass in small for gestational age preterm infants after term. PLoS ONE.

[21] American Academy of Pediatrics Committee on Nutrition (1985). Nutritional needs of low-birth-weight infants. Pediatrics.

[22] Ziegler E.E., Thureen P.J., Carlson S.J. (2002). Aggressive nutrition of the very low birthweight infant. Clin. Perinatol..

[23] Lubchenco L.O., Hansman C., Boyd E. (1966). Intrauterine growth in length and head circumference as estimated from live births at gestational ages from 26 to 42 weeks. Pediatrics.

[24] Ramel S.E., Demerath E.W., Gray H.L., Younge N., Boys C., Georgieff M.K. (2012). The relationship of poor linear growth velocity with neonatal illness and two-year neurodevelopment in preterm infants. Neonatology.

[25] Schmelzle H.R., Quang D.N., Fusch G., Fusch C. (2007). Birth weight categorization according to gestational age does not reflect percentage body fat in term and preterm newborns. Eur. J. Pediatr..

[26] Miller H.C., Hassanein K. (1971). Diagnosis of impaired fetal growth in newborn infants. Pediatrics.

[27] Paviotti G., Monasta L., Ronfani L., Montico M., Copertino M., De Cunto A., Demarini S. (2016). Body mass index curves for Italian preterm infants are comparable with American curves for infants born before 34 weeks of gestational age. Acta Paediatr..

[28] Cooke R.J., Griffin I. (2009). Altered body composition in preterm infants at hospital discharge. Acta Paediatr..

[29] Tudehope D., Fewtrell M., Kashyap S., Udaeta E. (2013). Nutritional needs of the micropreterm infant. J. Pediatr..

[30] Johnson M.J., Wootton S.A., Leaf A.A., Jackson A.A. (2012). Preterm birth and body composition at term equivalent age: A systematic review and meta-analysis. Pediatrics.

[31] Rochow N., Landau-Crangle E., Fusch C. (2015). Challenges in breast milk fortification for preterm infants. Curr. Opin. Clin. Nutr. Metab. Care.

[32] Kashyap S., Ohira-Kist K., Abildskov K., Towers H.M., Sahni R., Ramakrishnan R., Schulze K. (2001). Effects of quality of energy intake on growth and metabolic response of enterally fed low-birth-weight infants. Pediatr. Res..

[33] Andersen G.S., Girma T., Wells J.C., Kaestel P., Leventi M., Hother A.L., Michaelsen K.F., Friis H. (2013). Body composition from birth to 6 months of age in Ethiopian infants: Reference data obtained by air-displacement plethysmography. Am. J. Clin. Nutr..

[34] Villar J., Giuliani F., Bhutta Z.A., Bertino E., Ohuma E.O., Ismail L.C., Barros F.C., Altman D.G., Victora C., Noble J.A. (2015). Postnatal growth standards for preterm infants: The preterm postnatal follow-up study of the INTERGROWTH-21(st) project. Lancet Glob. Health.

