# Supplementary material for: Length Normalized Indices for Fat Mass and Fat-Free Mass in Preterm and Term Infants during the First Six Months of Life
Source: Nutrients. 2016 Jul 8;8(7):417. doi: 10.3390/nu8070417 (PMC4963893; doi:10.3390/nu8070417)
Supplement: Supplementary file 1 [file nutrients-08-00417-s001.docx]

Supplementary Materials: Length Normalized Indices for Fat Mass and Fat-Free Mass in Preterm and Term Infants during the First Six Months of Life

Ipsita Goswami, Niels Rochow, Gerhard Fusch, Kai Liu, Michael L. Marrin, Matthias Heckmann, Mathias Nelle and Christoph Fusch

**Table S1.** Length normalized indices in preterm infants.

| **PMA (Weeks)** | **Fat-Free Mass Index (kg/m^2^)** | | | | | | **Fat Mass Index(kg/m^2^)** | | | | | |
| --- | --- | --- | --- | --- | --- | --- | --- | --- | --- | --- | --- | --- |
|  | **3rd** | **10th** | **25th** | **50th** | **95th** | **97th** | **3rd** | **10th** | **25th** | **50th** | **95th** | **97th** |
| 28 | 5.2 | 5.9 | 6.8 | 7.8 | 11.1 | 11.7 | 0.1 | 0.1 | 0.1 | 0.1 | 1.2 | 1.9 |
| 30 | 5.8 | 6.6 | 7.4 | 8.4 | 11.4 | 11.9 | 0.1 | 0.2 | 0.3 | 0.4 | 2 | 2.6 |
| 32 | 6.5 | 7.3 | 8.1 | 9 | 11.7 | 12.2 | 0.2 | 0.3 | 0.5 | 0.8 | 2.5 | 3 |
| 34 | 7.2 | 8 | 8.7 | 9.6 | 12.1 | 12.5 | 0.3 | 0.5 | 0.7 | 1.1 | 3.1 | 3.5 |
| 36 | 7.8 | 8.5 | 9.3 | 10.2 | 12.5 | 12.8 | 0.4 | 0.7 | 1 | 1.6 | 3.7 | 4.1 |
| 38 | 8.2 | 8.9 | 9.7 | 10.5 | 12.7 | 13.1 | 0.6 | 0.9 | 1.4 | 2 | 4.2 | 4.6 |
| 40 | 8.4 | 9.2 | 9.9 | 10.8 | 13 | 13.4 | 0.8 | 1.2 | 1.7 | 2.5 | 4.8 | 5.2 |
| 42 | 8.6 | 9.4 | 10.1 | 11 | 13.3 | 13.7 | 1 | 1.5 | 2.1 | 2.9 | 5.4 | 5.8 |
| 44 | 8.8 | 9.6 | 10.3 | 11.2 | 13.6 | 14 | 1.1 | 1.8 | 2.4 | 3.3 | 5.8 | 6.2 |
| 46 | 8.9 | 9.7 | 10.5 | 11.4 | 13.8 | 14.2 | 1.3 | 2 | 2.7 | 3.6 | 6.1 | 6.5 |
| 48 | 8.9 | 9.7 | 10.5 | 11.4 | 13.9 | 14.3 | 1.5 | 2.2 | 3 | 3.9 | 6.4 | 6.7 |
| 50 | 8.9 | 9.7 | 10.5 | 11.4 | 13.9 | 14.4 | 1.7 | 2.4 | 3.2 | 4.1 | 6.6 | 6.9 |
| 52 | 8.9 | 9.7 | 10.5 | 11.4 | 13.9 | 14.4 | 1.9 | 2.6 | 3.4 | 4.3 | 6.7 | 7.1 |
| 54 | 8.9 | 9.7 | 10.5 | 11.4 | 13.9 | 14.4 | 2 | 2.8 | 3.6 | 4.5 | 6.9 | 7.2 |
| 56 | 8.9 | 9.7 | 10.5 | 11.4 | 13.9 | 14.4 | 2.2 | 2.9 | 3.7 | 4.6 | 7 | 7.3 |
| 58 | 8.9 | 9.7 | 10.5 | 11.4 | 13.9 | 14.4 | 2.3 | 3 | 3.8 | 4.7 | 7 | 7.4 |
| 60 | 8.9 | 9.7 | 10.5 | 11.4 | 13.9 | 14.4 | 2.5 | 3.1 | 3.9 | 4.7 | 7.1 | 7.4 |
| 62 | 8.9 | 9.7 | 10.5 | 11.4 | 13.9 | 14.4 | 2.6 | 3.3 | 4 | 4.8 | 7.1 | 7.4 |
| 64 | 9 | 9.7 | 10.5 | 11.4 | 13.9 | 14.3 | 2.8 | 3.4 | 4 | 4.8 | 7.1 | 7.4 |
| 66 | 9 | 9.7 | 10.5 | 11.4 | 13.8 | 14.3 | 2.9 | 3.5 | 4.1 | 4.9 | 7 | 7.4 |
| 68 | 9 | 9.7 | 10.5 | 11.3 | 13.7 | 14.1 | 3.1 | 3.6 | 4.2 | 4.9 | 7 | 7.3 |
| 70 | 9 | 9.7 | 10.5 | 11.3 | 13.5 | 13.9 | 3.3 | 3.7 | 4.3 | 4.9 | 6.9 | 7.2 |

**Table S2.** Length normalized indices in term infants.

| **PMA (Weeks)** | **Fat-Free Mass Index (kg/m^2^)** | | | | | | **Fat Mass Index (kg/m^2^)** | | | | | |
| --- | --- | --- | --- | --- | --- | --- | --- | --- | --- | --- | --- | --- |
|  | **3rd** | **10th** | **25th** | **50th** | **95th** | **97th** | **3rd** | **10th** | **25th** | **50th** | **95th** | **97th** |
| 38 | 8.9 | 9.4 | 9.9 | 10.4 | 12.3 | 12.7 | 0.7 | 0.9 | 1.1 | 1.4 | 3.1 | 3.6 |
| 40 | 9.2 | 9.7 | 10.2 | 10.8 | 12.7 | 13 | 0.9 | 1.1 | 1.3 | 1.7 | 3.4 | 3.8 |
| 42 | 9.3 | 9.9 | 10.4 | 11 | 12.9 | 13.2 | 1 | 1.3 | 1.6 | 2 | 3.7 | 4.1 |
| 44 | 9.4 | 9.9 | 10.5 | 11.1 | 13 | 13.4 | 1.3 | 1.6 | 2 | 2.6 | 4.4 | 4.8 |
| 46 | 9.4 | 10 | 10.5 | 11.2 | 13.1 | 13.4 | 1.6 | 2 | 2.5 | 3.2 | 5.2 | 5.6 |
| 48 | 9.4 | 10 | 10.6 | 11.2 | 13.1 | 13.5 | 1.9 | 2.4 | 3 | 3.8 | 6.1 | 6.5 |
| 50 | 9.3 | 9.9 | 10.5 | 11.2 | 13.1 | 13.5 | 2.2 | 2.8 | 3.5 | 4.3 | 6.8 | 7.3 |
| 52 | 9.1 | 9.8 | 10.4 | 11.1 | 13 | 13.4 | 2.5 | 3.2 | 3.9 | 4.7 | 7.4 | 7.9 |
| 54 | 9 | 9.7 | 10.3 | 11 | 12.9 | 13.3 | 2.7 | 3.4 | 4.2 | 5 | 7.7 | 8.1 |
| 56 | 8.8 | 9.6 | 10.2 | 11 | 12.9 | 13.2 | 2.9 | 3.6 | 4.3 | 5.2 | 7.8 | 8.2 |
| 58 | 8.7 | 9.5 | 10.2 | 10.9 | 12.8 | 13.2 | 3.2 | 3.8 | 4.5 | 5.4 | 7.9 | 8.4 |
| 60 | 8.7 | 9.4 | 10.1 | 10.9 | 12.8 | 13.1 | 3.4 | 4 | 4.7 | 5.5 | 8 | 8.5 |
| 62 | 8.6 | 9.4 | 10.1 | 10.8 | 12.8 | 13.1 | 3.6 | 4.3 | 4.9 | 5.7 | 8.2 | 8.7 |
| 64 | 8.5 | 9.3 | 10 | 10.7 | 12.7 | 13 | 3.9 | 4.5 | 5.2 | 6 | 8.5 | 8.9 |
| 66 | 8.3 | 9.1 | 9.8 | 10.6 | 12.4 | 12.7 | 4.2 | 4.8 | 5.4 | 6.2 | 8.8 | 9.3 |
